# Rich Oxygen Vacancies in Bimetallic MnCo_2_O_4.5_ Spheres for Enhancing Lean Methane Catalytic Oxidation

**DOI:** 10.3390/nano15070524

**Published:** 2025-03-31

**Authors:** Ke Yang, Chenqi Li, Qinghan Zhu, Haiwang Wang, Jian Qi

**Affiliations:** 1 Key Laboratory of Dielectric and Electrolyte Functional Material Hebei Province, Northeastern University at Qinhuangdao, Qinhuangdao 066004, China; 2272412@stu.neu.edu.cn (K.Y.); 2372207@stu.neu.edu.cn (C.L.); 2272429@stu.neu.edu.cn (Q.Z.); 2 State Key Laboratory of Biochemical Engineering, Institute of Process Engineering, Chinese Academy of Sciences, Beijing 100190, China; 3 School of Chemical Engineering, University of Chinese Academy of Sciences, Beijing 100049, China

**Keywords:** MnCo_2_O_4.5_ spheres, bimetallic oxide, oxygen vacancies, hierarchical structure, methane combustion

## Abstract

Methane is the second most prevalent greenhouse gas after carbon dioxide in global climate change, and catalytic oxidation technology is a very effective way to eliminate methane. However, the high reaction temperature of methane catalytic oxidation is an urgent problem that needs to be solved. In this work, a series of MnCo_2_O_4.5_ catalysts were prepared using carbon spheres as templates, combined with metal ion adsorption and calcination processes. Excitingly, the catalytic oxidation activity of MnCo_2_O_4.5_ spherical catalyst with irregular nanoparticles on the surface for lean methane (T_90_ = 395 °C) is higher than that of pure phase Co_3_O_4_ (T_90_ = 538 °C) and Mo_3_O_4_ (T_90_ = 581 °C) spherical catalysts and even surpasses most precious metal catalysts. The main reasons are as follows: (1) The spherical core with irregular nanoparticle morphology significantly increases the specific surface area, creating abundant active sites; (2) through the optimized distribution of oxygen vacancies, rapid oxygen migration through this structure can quickly enter the catalytic zone; (3) the hierarchical wall structure expands the interface and provides spatial accommodation for the catalytic process. Meanwhile, the structure of the ball wall further expands the reaction interface, providing sufficient space for the occurrence of reactions. Rich and highly active oxygen vacancies are evenly distributed on the surface and inside of the ball. The extraordinary performance of low-temperature methane combustion catalysts has opened a promising new path, which is expected to inject strong impetus into the global energy transition and environmental protection.

## 1. Introduction

In the contemporary era, the intertwined challenges of energy shortage and environmental pollution have propelled scientific research towards seeking sustainable solutions. Methane, a principal component of natural gas, has emerged as a crucial subject of investigation due to its dual nature [1,2,3]. On the one hand, it represents a valuable energy resource; on the other, as a potent greenhouse gas with a global warming potential approximately 25 times greater than carbon dioxide over a 100-year time scale, its uncontrolled release into the atmosphere poses a severe threat to the global climate [4,5,6]. Notably, vast amounts of low-concentration methane are generated from diverse sources such as coal mine gas, landfill gas, and biogas from agricultural waste, where the methane content typically ranges from a few percent to around 30% [7,8]. Efficient utilization of this low-concentration methane not only mitigates greenhouse gas emissions but also unlocks an alternative energy reservoir, thereby holding the key to harmonizing energy demands with environmental preservation.

As is well known, the methane content in coalbed methane is about 95%, but it is often mixed with air after extraction, forming low concentrations of methane (such as 0.75%). Direct emissions can lead to the greenhouse effect. Therefore, catalytic oxidation has become a key emission reduction technology. By leveraging catalytic action, methane can be oxidized to carbon dioxide and water at relatively low temperatures, achieving both energy recovery and emission reduction [9]. Central to this process is the development of highly efficient catalysts. Transition metal oxides, with their rich redox properties, adjustable electronic structures, and cost-effectiveness compared with noble metals, have garnered significant attention in recent years [10,11]. The study showed that high concentrations of methane (such as >16%) can cause severe heat release, and the catalyst may become deactivated due to high-temperature sintering. Low concentrations (such as <1%) result in insufficient heat release during the reaction, leading to unstable surface temperature of the catalyst and passivation of active sites.

However, traditional single-metal catalysts, such as noble metals Pd [12] and Pt [13] or transition metal oxides like Co_3_O_4_ and Mn_3_O_4_ [4,14], commonly suffer from limitations, including a narrow active temperature window, deactivation due to high-temperature sintering, and inadequate resistance to sulfur and water [9,15]. These issues significantly restrict their large-scale application. Taking Pd-based bimetallic catalysts as an example, despite their excellent low-temperature oxidation performance, the high cost, susceptibility to spinning at high temperatures, and insufficient stability in complex industrial environments remain major challenges [12,16,17]. Against this backdrop, bimetallic catalysts have become a research hotspot in recent years due to their unique electronic synergistic effects, adjustable active sites, and enhanced stability.

For instance, Liu et al. confirmed through in situ XPS that the Mn-Co synergy could increase the proportion of surface oxygen species (O^-^, O^2-^) by 40%, thereby reducing the ignition temperature of methane by 16% compared with single Co_3_O_4_ catalysts, demonstrating significant advantages [18]. Additionally, Du. et al. utilized DFT calculations to reveal the charge redistribution phenomenon at the Mn-Co bimetallic interface, where electron transfer reduced the activation energy barrier for breaking methane C-H bonds by 0.3 eV, further supporting the activity enhancement mechanism of the bimetallic system theoretically [19].

Nevertheless, most existing studies focus on the synthesis and performance optimization of powdered MnCo_2_O_4.5_, whose disordered stacking structure leads to shielding of active sites, increased mass transfer resistance, and insufficient mechanical strength [20,21,22]. This is especially problematic under high temperature and high flow rate conditions, where particle agglomeration and pore blockage can occur, severely limiting practical application potential.

To address these issues, this work innovatively designed and prepareed MnCo_2_O_4.5_ catalysts with a hierarchical structure featuring a spherical core-nanoparticle shell. The aim was to achieve synergistic improvements in activity, stability, and engineering applicability through morphology engineering and interface regulation [23,24]. The spherical core structure provides high mechanical strength and regular diffusion channels, while ultrafine nanoparticles attached to the surface maximize the exposure of active sites and shorten the mass transfer path of reactants [25,26]. This design concept draws inspiration from successful cases of porous core-shell materials in catalysis. Wang et al. synthesized a core-shell flower-like cobalt-manganese composite catalyst via a hydrothermal method by substituting Co^2+^ with Mn^2+^. This approach effectively leveraged the advantages of carrier materials, unique morphology, and doping modification, thereby reducing the sintering-induced deactivation risk of Co_3_O_4_ while demonstrating enhanced catalytic activity. Compared with Co_3_O_4_/SiO_2_, the methane conversion rates of the composite catalyst increased by 10% and 6% at 350 °C and 450 °C, respectively [27]. Ma et al. developed the Pd (PdO)/Co_3_O_4_@SiO_2_ bimetallic oxide core-shell catalyst via a template-assisted method. This study revealed that the bimetallic oxide core-shell structure effectively strengthens the metal-metal interaction between Pd and Co, thereby weakening the Co-O bond strength in Pd (PdO)/Co_3_O_4_@SiO_2_. The weakened Co-O bonds facilitate the release of more lattice oxygen, which participates in CH₄ activation, endowing the catalyst with superior catalytic performance in low-temperature methane combustion [28]. These studies provide important references for the application of spherical structures in catalysis, but systematic reports on the hierarchical structural design of the Mn-Co bimetallic system and its targeted regulation mechanism for methane combustion are still lacking [29,30,31]. Specifically, the size effect of nanoparticles on the shell, interfacial electronic states, and the relationship between methane oxidation kinetics and structure-performance have not been clarified.

The scientific significance of this study lies in overcoming the performance bottlenecks of bimetallic catalysts with spherical core-nanoparticle shells through cross-scale structural design. Specifically, the spherical core can be uniformly sized using template methods, and its internal porous network promotes rapid diffusion of methane molecules. Uniformly distributed MnCo_2_O_4.5_ nanoparticles on the surface offer high-density active sites. This structure yields several advantages: (1) the high surface energy of nanoparticles enhances methane adsorption capacity, and the small size effect promotes lattice oxygen activation; (2) the mechanical support provided by the spherical core inhibits nanoparticle migration at high temperatures, alleviating sintering phenomena; and (3) charge transfer at the core-shell interface further optimizes the bimetallic synergistic effect.

## 2. Experimental

### 2.1. Materials and Chemicals

The reagents were not subjected to further purification. Cobalt nitrate hexahydrate (Co(NO_3_)_2_·6H_2_O) and manganese acetate (Mn(CH_3_COO)_2_·4H_2_O) were purchased from Aladdin Reagent Co. (Fengxian District, Shanghai city, China) Urea (CO(NH_2_)_2_) was obtained from Sinopharm Chemical Reagent Co (Xicheng District, Beijing city, China), and sucrose (C_12_H_22_O_11_) was sourced from McLean Reagent C (Pudong New Area, Shanghai city, China).

### 2.2. Preparation of Carbon Spheres

A total of 16.00 g of sucrose was dissolved in 160 mL of water. The resultant mixture was then transferred into a sealed Teflon autoclave and crystallized continuously for 8 h at 180 °C. After crystallization, the black product was isolated from the mixture via centrifugation at 8000 rpm for 3 min. Subsequently, the separated black product was rinsed multiple times with water and anhydrous alcohol to eliminate residual impurities. Eventually, the washed product was dried in an oven at 80 °C for 12 h.

### 2.3. Preparation of MnCo_2_O_4.5_ Catalysts

**Scheme 1:**
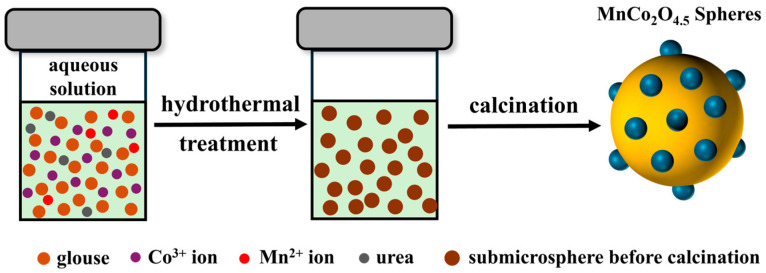
Schematic illustration depicts the formation processes of the MnCo_2_O_4.5_ spheres catalyst.

Synthesis of MnCo_2_O_4.5_ bimetallic oxide catalyst: A mixture of 0.60 g sucrose carbon spheres, 0.49 g manganese acetate, 2.91 g cobalt nitrate, and 3.00 g urea were dissolved in a solution of 15 mL water and 45 mL ethanol, then ultrasonicated for 15 min. The suspension was heated in a Teflon-lined autoclave at 80 °C for 6 h, followed by washing with deionized water and ethanol and drying overnight at 80 °C. The dried precursor was then calcined in air at 500 °C for 1 h with a heating rate of 1 °C/min. By adjusting the amounts of cobalt nitrate and manganese acetate, composites with Mn/Co molar ratios of 1:4, 1:5, 1:6, and 1:7 were synthesized. The preparation procedure of the MnCo_2_O_4.5_ bimetallic oxide catalyst was depicted in Scheme 1. The synthesis of Co_3_O_4_ and Mn_3_O_4_ catalysts is detailed in Text S1.

### 2.4. Characterization

The catalysts were analyzed through X-ray diffraction (XRD) using a DX-2500 X-ray diffractometer (equipped with a Cu-Kα radiation source, λ = 0.15406 nm, with a maximum output power of 9 kW), with the scanning angle range set from 20° to 70° and the scanning speed maintained at 5 °/min. The Brunner–Emmett–Taylor (BET) surface area of the catalysts was accurately measured using an SSA-4000 specific surface area analyzer (with a built-in MD-200 sample preprocessor and a preheating procedure of 180 min at 120 °C). The catalyst was rigorously degassed prior to measurement to ensure the accuracy of the results. The overall micro-morphology of the catalyst was observed using a German Supra 55 Scanning Electron Microscope (SEM). The micro-morphology and crystal structure of the samples were observed using a Philips-FEI high-resolution transmission electron microscope (Tecnai G2 F20, TEM). X-ray photoelectron spectroscopy (XPS) analysis was carried out using an Escalab 250 X-ray photoelectron spectrometer, and the resulting binding energies were calibrated by the characteristic peak of C 1s at 284.8 eV to reveal the chemical state of the catalyst surface. Raman spectroscopy measurements were performed on a spectrometer using a 532 nm diode laser as the excitation source to further probe the molecular vibration and rotation information of the catalyst. Hydrogen Temperature-Programmed Reduction (H_2_-TPR) and Oxygen Temperature-Programmed Desorption (O_2_-TPD) experiments were performed using the Micromeritics AutoChem II 2920 instrument. In the H_2_-TPR experiment, He gas was first passed at a flow rate of 50 mL/min for 60 min to purify the system, followed by switching to a 5 vol% H_2_/Ar gas mixture at 50 mL/min and heating rate of 10 °C/min from room temperature to 800 °C. The reduction behavior of the catalyst was observed and recorded. The O_2_-TPD experiment, on the other hand, followed a similar procedure, but the gas was replaced with a 50 mL/min 5 vol% O_2_/He gas mixture to probe the oxygen adsorption and desorption characteristics of the catalyst.

### 2.5. Catalytic Activity Testing

The methane catalytic oxidation tests were conducted in a quartz fixed-bed reactor (5 mm inner diameter, 550 mm length). A 100 mg catalyst sample was positioned centrally within the reactor to optimize gas-catalyst contact. The reactant feed comprised 1 vol% CH_4_, 20 vol% O_2_, and 0–10 vol% H_2_O (if applicable), balanced with N_2_, and maintained at a gas hourly space velocity (GHSV) of 36,000 mL·g⁻^1^·h⁻^1^. Temperature control was achieved using a resistance furnace equipped with an integrated thermocouple.

First, the reactor was heated from room temperature to 200 °C within 10 min. After reaching 200 °C, the system maintained this temperature for 5 min to ensure the stability of the catalyst. Subsequently, the temperature was increased by 25 °C every 10 min until the catalytic process was complete. Throughout the entire temperature increase process, samples were taken at each temperature point to monitor the changes in methane conversion efficiency.

The conversion of methane was determined using the following equation:

$${\mathrm{CH}}_{4}\left(\%\right)=\frac{[{{\mathrm{CH}}_{4}]}_{\mathrm{in}}-[{\mathrm{CH}}_{4}{]}_{\mathrm{out}}}{[{{\mathrm{CH}}_{4}]}_{\mathrm{in}}}\times 100\%,$$

where *[CH_4_]_in_* represents the initial methane concentration, and *[CH_4_]_out_* represents the methane concentration after catalytic oxidation.

In chromatographic quantitative analysis, peak areas can be utilized for calculation. Both the content of components in the sample and their corresponding peak areas must exhibit a linear relationship. The calculation procedure for determining methane concentration using the external standard method is as follows:

Calculation of Absolute Correction Factor ($g_{\left[CH_{4}\right]}$):

$$g_{\left[CH_{4}\right]}=\frac{C_{\left[CH_{4}\right]}}{A_{\left[CH_{4}\right]}},$$

where $C_{\left[CH_{4}\right]}$ represents the methane content in the 1% CH_4_ standard sample, and $A_{\left[CH_{4}\right]}$ denotes the peak area of methane in the chromatogram of the 1% CH_4_ standard sample.

Quantitative Formula for External Standard Method:

$$C_{i}=A_{\left[CH_{4}\right]}\times g_{\left[CH_{4}\right]}$$

where *C_i_* is the methane content in the unknown sample.

This method establishes a proportional relationship between the peak area of the target component and its concentration based on the calibration factor derived from the standard sample.

## 3. Results and Discussion

### 3.1. Physicochemical Properties

**Figure 1:**
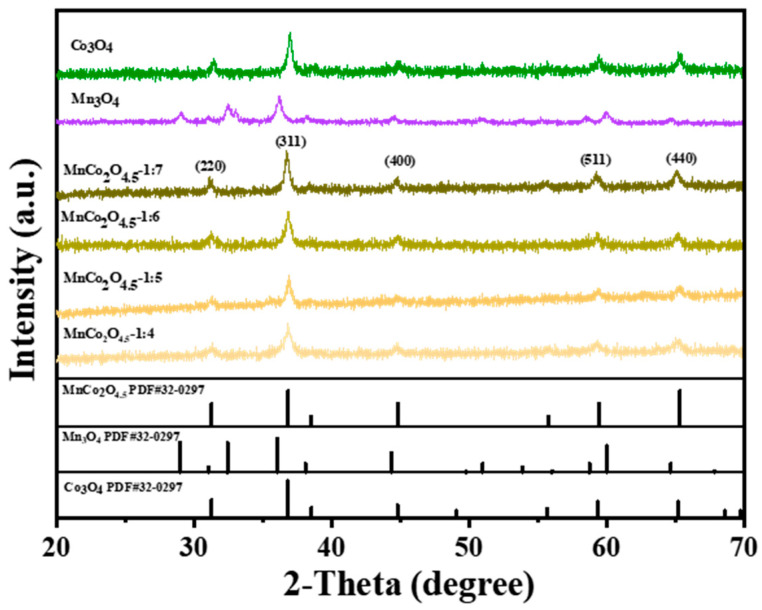
XRD patterns of different samples: MnCo_2_O_4.5_-1:4, MnCo_2_O_4.5_-1:5, MnCo_2_O_4.5_-1:6, MnCo_2_O_4.5_-1:7, Mn_3_O_4_, and Co_3_O_4_.

The crystal structure of the samples was comprehensively analyzed using X-ray diffraction (XRD). Figure 1 shows the XRD patterns of samples with different Mn/Co molar ratios. Obvious diffraction peaks were observed at 2θ = 18.98°, 36.82°, 44.83°, 59.47°, and 65.34°, corresponding to the (220), (311), (400), (511), and (440) crystal planes, respectively. These peaks are consistent with the standard MnCo_2_O_4.5_ phase (PDF#32-0297), confirming the successful formation of the structure. As the Mn/Co molar ratio changes from 1:4 to 1:7, the full width at half maximum (FWHM) of the XRD peak gradually narrows, and the crystallinity increases. At the same time, the diffraction intensity increases, and the preferred orientation is enhanced. This phenomenon is attributed to the higher bond strength of Co-O (~242 kJ/mol) compared with Mn-O (~206 kJ/mol), which promotes preferential growth along the (311) plane in the cobalt-rich composition [32,33]. In the figure, Mn_3_O_4_ (PDF#18-0803) and Co_3_O_4_ (PDF#74-2120) were successfully indexed, and no diffraction peaks of other phases were observed, confirming the absence of impurity phases in the synthesized sample. Figure S1. indicates that extending the calcination time can improve crystalline, resulting in clearer and stronger XRD diffraction peaks.

**Figure 2:**
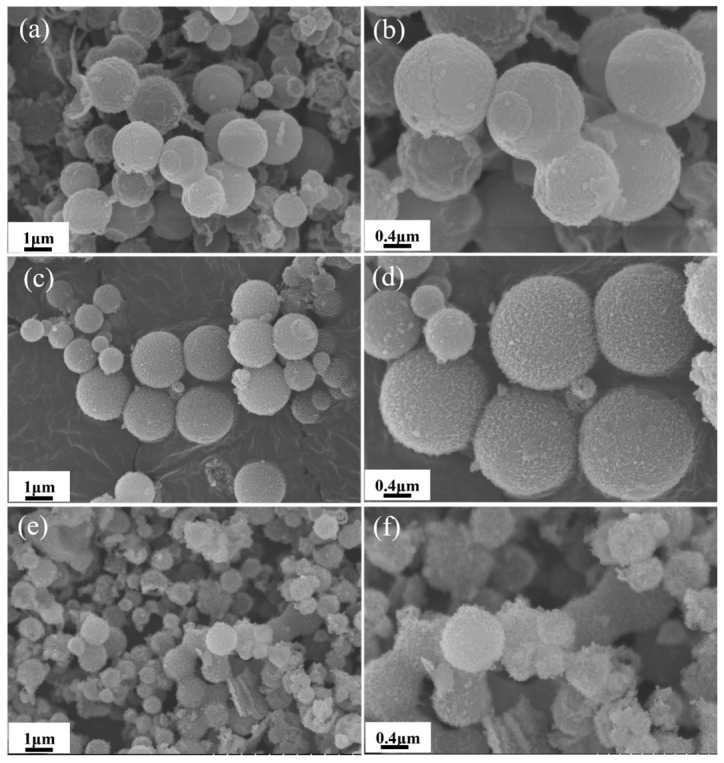
(**a**,**b**) SEM images of MnCo_2_O_4.5_-1:5. (**c**,**d**) SEM images of Co_3_O_4_. (**e**,**f**) SEM images of Mn_3_O_4_.

Scanning Electron Microscopy (SEM) was utilized to systematically investigate the morphological and structural characteristics of the materials, aiming to elucidate the impact of Mn and Co bimetallic ion incorporation on the formation of bimetallic oxides. As illustrated in Figure 2a,b, the MnCo_2_O_4.5_-1:5 sample exhibits a distinctive spherical core-nanoparticle shell hierarchical structure. The micro-sized spherical cores (approximately 1–3 μm in diameter) are uniformly coated with ultrafine nanoparticles, creating a high-density active interface. This hierarchical morphology can increase the specific surface area, providing more active sites for methane absorption and oxygen species activation. Additionally, the regular pore network within the spherical cores can shorten the diffusion paths of reactants and reduce mass transfer resistance [3,34].

Moreover, the small size of the nanoparticles facilitates lattice oxygen activation, with their highly exposed crystal facets rich in unsaturated coordinated atoms being more prone to methane adsorption and C-H bond cleavage [18]. In contrast, as shown in Figure 2c,d, pure Co_3_O_4_ presents spherical morphology with smooth and dense surfaces, low porosity, and limited exposure to active sites. Pure Mn_3_O_4_ predominantly forms irregular aggregates with a broad distribution of particle sizes, leading to partial pore blockages, lower specific surface areas, and inferior mass transfer efficiency compared with the bimetallic system.

The notable morphological evolution observed in MnCo_2_O_4.5_ indicated that Mn incorporation played a critical role in promoting the formation of larger, structurally intact spherical particles. This phenomenon may be attributed to manganese-induced nucleation kinetics and interfacial energy regulation during synthesis, which favor controlled particle growth and surface nanostructuring. Such insights into the synergistic effects between Mn and Co provide valuable understanding for optimizing the design of catalysts for efficient catalytic reactions. Figure S2 illustrates the SEM morphology evolution of MnCo_2_O_4.5_ catalysts subjected to different calcination times ranging from 0.5 h to 3 h, highlighting the critical role of calcination duration in regulating the microstructure of bimetallic catalysts and its mechanism affecting catalytic performance. The MnCo_2_O_4.5_-0.5 h sample (Figure S2a,b) shows an initial nanoparticle aggregation structure with uneven distribution and noticeable uncrystallized regions on the surface, indicating insufficient crystal growth due to short calcination time. The pore network is not fully developed at this stage.

In contrast, the MnCo_2_O_4.5_-1 h sample (Figure S2c,d) exhibits an optimized hierarchical morphology: nanoparticles are densely packed into a porous network with regular pore distribution. This structure strikes a balance between crystalline and porosity, providing ideal pathways for methane molecule adsorption and oxygen species migration. However, the calcination time extends to 2 h (Figure S2e,f) and 3 h (Figure S2g,h), and significant morphological degradation occurs. The MnCo_2_O_4.5_-2 h sample begins to show dense agglomerates in localized areas with a reduced number of pores. Further extending the calcination time to 3 h results in extensive sintering phenomena in the MnCo_2_O_4.5_-3 h sample, where particle boundaries become blurred and pores are almost completely blocked. This leads to a severe loss of active interfaces. The calcination process appears to be dominated by the Ostwald ripening mechanism, ultimately causing size inhomogeneity and pore blockage. At high temperatures, the framework structure contracts, leading to the closure of original pore channels and obstruction of mass transfer paths.

**Figure 3:**
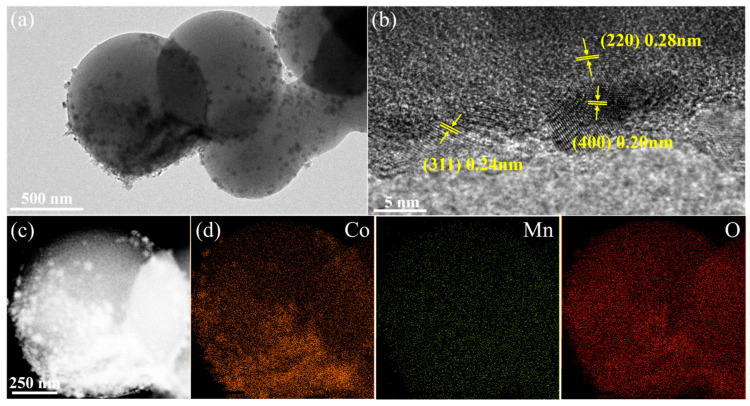
(**a**) TEM image of MnCo_2_O_4.5_-1:5; (**b**) HRTEM image of MnCo_2_O_4.5_-1:5; (**c**) High-angle circular dark field image (HAADF) of MnCo_2_O_4.5_-1:5 and (**d**) EDS Mapping of corresponding particles.

The crystal structure and elemental distribution of the MnCo_2_O_4.5_ catalyst were systematically investigated using transmission electron microscopy (TEM). As illustrated in Figure 3a, the catalyst shows uniformly dispersed spherical particles (diameter: 1~3 μm) decorated with protruding irregular nanoparticles (30~50 nm). High-resolution TEM imaging (Figure 3b) reveals well-defined lattice fringes with measured spacings of approximately 0.20 nm, 0.24 nm, and 0.28 nm, corresponding to the (400), (311), and (220) crystallographic planes of spinel-structured MnCo_2_O_4.5_ (PDF#32-0297), respectively. Figure 3c confirms the sample with spherical core-nanoparticle shell structures. Elemental mapping (Figure 3d) confirms the homogeneous distribution of Co, Mn and O within the spherical matrix. The dual-functional surface nanostructure represents a novel strategy for preventing particle agglomeration during high-temperature reactions while maintaining long-term operational stability [28].

**Figure 4:**
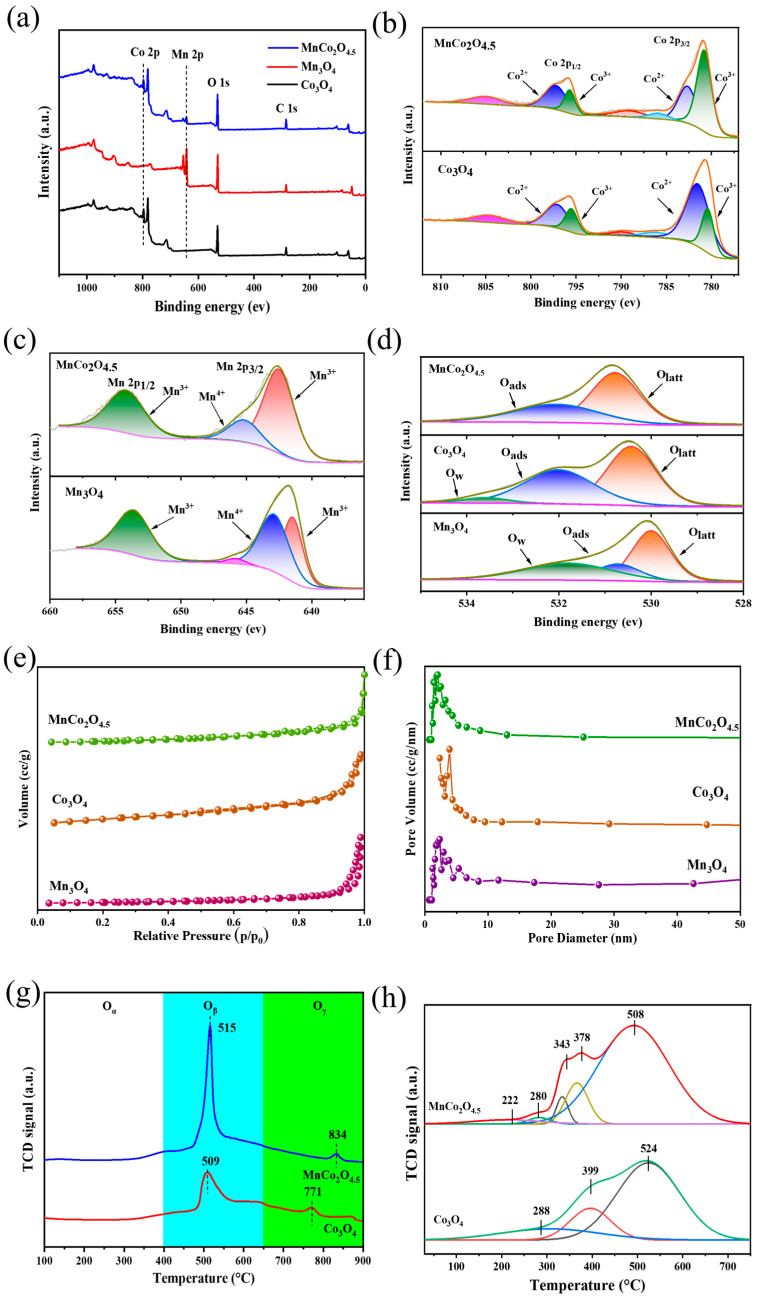
(**a**) XPS full-scan spectra; (**b**) Co 2p energy level spectra; (**c**) Mn 2p energy level spectra; (**d**) O 1s energy level spectra; (**e**) N_2_ adsorption-desorption curves and (**f**) pore size distributions of different samples; (**g**) O_2_-TPD profiles; and (**h**) H_2_-TPR profiles of MnCo_2_O_4.5_ and Co_3_O_4_.

X-ray photoelectron spectroscopy (XPS) was employed to determine the surface composition and chemical states of the catalyst, providing insights into its electronic structure. Figure 4a presents the XPS survey spectra of all samples, confirming the elemental composition of the catalysts. The spectra reveal distinct peaks corresponding to Co, Mn, O, and adventitious carbon (C 1s at ~285 eV). Figure 4b displays the Co 2p spectrum, showing the spin-orbit splitting of the 2p_1/2_ and 2p_3/2_ levels due to spin-orbit coupling effects. The primary peaks are located at approximately 781 eV (Co 2p_3/2_) and 796 eV (Co 2p_1/2_), each further split into sub-peaks corresponding to Co^3+^ and Co^2+^, indicating the coexistence of Co in mixed +2 and +3 oxidation states [35,36,37]. Additionally, satellite peaks observed at ~788 eV and ~804 eV further confirm this mixed-valence nature. Figure 4c presents the XPS spectrum of MnCo_2_O_4.5_, which exhibits fitted Mn 2p peaks. The Mn 2p_3/2_ and Mn 2p_1/2_ peaks are located at ~643 eV and ~654 eV, respectively, which is attributed to the presence of Mn^3+^ and Mn^4+^ oxidation states [18,38]. Mn^3+^ ions, occupying an intermediate valence state, exhibit excellent electron-switching capability, enabling smooth transitions between different Mn oxidation states. This characteristic significantly will be instrumental in enhancing redox efficiency in catalytic reactions, promoting oxygen mobility and facilitating lattice oxygen involvement in methane activation. Furthermore, the formation of Mn^3+^ is often associated with the generation of highly active surface-adsorbed oxygen species, which will play a crucial role in gas-phase oxidation reactions, further accelerating the overall catalytic process.

The catalytic efficiency of the catalyst is significantly influenced by its surface chemical states, redox properties, and the nature and abundance of surface oxygen species. XPS analysis indicates that MnCo_2_O_4.5_ and Co_3_O_4_ exhibit highly similar Co 2p spectra, characterized by two distinct peaks corresponding to the spin-orbit splitting of the Co 2p_3/2_ and Co 2p1/2 orbitals. By evaluating the Co^3+^/Co^2+^ ratio (Table 1), it is observed that the surface Co^3+^/Co^2+^ ratio of MnCo2O4.5 (1.21) exceeds that of Co3O4 (0.41), suggesting that the coexistence of multiple oxidation states in the bimetallic oxide induces the formation of additional oxygen vacancies. Previous studies have demonstrated that a higher Co^3+^ content facilitates greater methane adsorption, which is critical for redox activity in catalytic cycles, thereby will be favor of enhancing methane conversion efficiency.

As shown in Figure 4c, MnCo_2_O_4.5_ and Mn_3_O_4_ exhibit nearly identical Mn 2p spectra. The calculated Mn^3+^/Mn^4+^ ratio (Table 1) reveals that MnCo_2_O_4.5_ has a higher surface Mn^3+^/Mn^4+^ ratio (5.91) compared with Mn_3_O_4_ (1.66), indicating that Mn ions predominantly exist as Mn^3+^ species. Mn^3+^ is identified as the primary active site for catalytic methane oxidation. In addition, O 1s XPS spectra were employed to investigate surface oxygen species. Figure 4d illustrates the O 1s spectra of each catalyst, where peaks at approximately 530 eV and 532 eV correspond to surface-adsorbed oxygen (O_ads_) and lattice oxygen (O_latt_), respectively [39,40]. A higher O_ads_/O_latt_ ratio signifies an abundance of reactive oxygen species, which is favorable for methane oxidation. Collectively, these spectroscopic analyses provide valuable insights into the electronic and structural aspects of MnCo_2_O_4.5_, offering a deeper understanding of the factors governing its superior catalytic performance.

N_2_ adsorption-desorption isotherms are crucial tools for assessing the specific surface area and pore structure of catalysts. As shown in Figure 4e, according to the classification by the International Union of Pure and Applied Chemistry (IUPAC), the materials obtained in this study exhibit characteristics of Type IV isotherms, which typically indicate the presence of mesoporous structures [41,42]. Additionally, the presence of hysteresis loops in the P/P_0_ range of 0.4 to 1.0 further confirms the existence of mesopores in MnCo_2_O_4.5_, Co_3_O_4_, and Mn_3_O_4_ samples. This phenomenon is especially evident in Type IV isotherms, characterized by a noticeable hysteresis loop resulting from capillary condensation occurring in mesopores. The pore size distribution curves derived from nitrogen desorption data (as shown in Figure 4f) further confirm the hierarchical mesoporous structures present in MnCo_2_O_4.5_, Co_3_O_4_, and Mn_3_O_4_ samples. As shown in Table S1, MnCo_2_O_4.5_ exhibits a BET-specific surface area of 56.58 m^2^/g, significantly higher than that of Co_3_O_4_ (41.14 m^2^/g) and Mn_3_O_4_ (13.09 m^2^/g). This result can be attributed to MnCo_2_O_4.5_ unique hierarchical porous structure, featuring spherical cores covered with nanoparticles on the surface. Such a structure not only increases the specific surface area but also provides more active sites. These features are critical for enhancing catalytic performance because a larger surface area means more reactants can interact with the catalyst’s active sites, thereby accelerating reaction rates.

Oxygen-temperature programmed desorption (O_2_-TPD) was employed to characterize the surface oxygen species and their desorption behavior. Typically, O_2_ desorption profiles can be classified into three regions (Figure 4g). The low-temperature region (< 450 °C) corresponds to chemisorbed oxygen (O_chem_), denoted as O_α_. The medium-temperature region (450–650 °C) is attributed to the desorption of surface lattice oxygen (O_surf_) and bulk lattice oxygen (O_bulk_), marked as O_β_. The high-temperature region (> 650 °C) represents lattice oxygen desorption or oxygen release from metal-oxygen bonds within the catalyst, designated as O_γ_ [36,43]. The O_2_-TPD profiles reveal that both MnCo_2_O_4.5_ and Co_3_O_4_ exhibit two desorption peaks. MnCo_2_O_4.5_ displays a more pronounced O_β_ peak, indicating enhanced lattice oxygen (O^2-^) release, which helps to increase catalytic activity at elevated temperatures. Furthermore, MnCo_2_O_4.5_ exhibits stronger low-temperature desorption, demonstrating superior oxygen activation capability. Quantitative analysis of oxygen desorption content across different catalysts confirms the enhanced oxygen storage and mobility in MnCo_2_O_4.5_, which is crucial for its superior catalytic performance in methane oxidation.

The reducibility of MnCo_2_O_4.5_ and Co_3_O_4_ catalysts was investigated via H_2_-TPR analysis. As shown in Figure 4h, five distinct reduction peaks were identified for MnCo_2_O_4.5_ through peak fitting. The first two peaks, observed at low temperatures, correspond to the reduction of surface-adsorbed oxygen or weakly bound oxygen species: the first peak at 222 °C is attributed to the reduction of Mn^3+^ → Mn^2+^, while the second peak at 280 °C is assigned to Co^3+^ → Co^2+^. The third and fourth peaks, which are observed at intermediate temperatures of 343 °C and 378 °C, are ascribed to the reduction of more stable lattice oxygen species. Specifically, the third peak corresponds to Co^2+^ → Co, and the fourth peak rises from Mn^4+^ → Mn^2+^. The fifth peak, located at 508 °C, represents the reduction in highly stable oxygen species or strongly bound metal ions assigned to CoO → Co. In contrast, Co_3_O_4_ exhibits three reduction peaks at 288 °C, 399 °C, and 524 °C, attributed to Co^3+^ → Co^2+^, Co^2+^ → Co, and CoO → Co, respectively [12,22,33,44].

Notably, all reduction peaks of MnCo_2_O_4.5_ occur at lower temperatures compared to those of Co_3_O_4_, indicating enhanced reducibility and higher catalytic activity, as lower reduction temperatures generally correlate with superior catalytic performance. Furthermore, the H₂ consumption of MnCo_2_O_4.5_ (18.57 μmol/g) significantly exceeds that of Co_3_O_4_ (15.09 μmol/g), demonstrating stronger reducibility and greater availability of active oxygen species in the bimetallic catalyst. Lower reduction temperatures and higher H₂ uptake collectively contribute to the improved catalytic activity of MnCo_2_O_4.5_ in methane combustion.

### 3.2. Performance of Catalytic Activity

**Figure 5:**
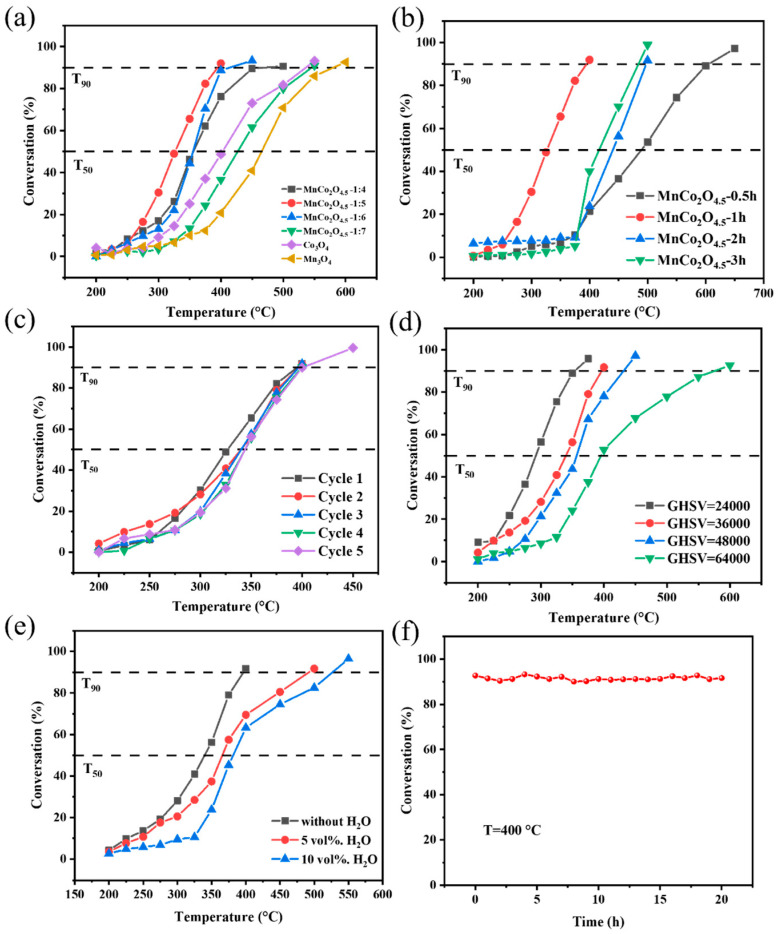
(**a**) Catalytic activity of MnCo_2_O_4.5_-1:4, MnCo_2_O_4.5_-1:5, MnCo_2_O_4.5_-1:6, MnCo_2_O_4.5_-1:7, Co_3_O_4_, and Mn_3_O_4_, gas mixer: 1 vol.% CH_4_, 20 vol.% O_2_, N_2_ as balance gas, GHSV = 36,000 mL·g^−1^·h^−1^; (**b**) cyclic test of MnCo_2_O_4.5_-1:5; (**c**) catalytic activity of MnCo_2_O_4.5_-1:5 with different calcination times with calcination temperature at 500 °C; (**d**) catalytic activity of MnCo_2_O_4.5_-1:5 at different water vapor; (**e**) catalytic activity of MnCo_2_O_4.5_-1:5 at different GHSVs; (**f**) stability evaluation of methane catalytic oxidation at 400 °C.

The catalytic performance of the synthesized catalysts was evaluated through methane oxidation reactions. The catalytic activity was quantified using T_90_ and T_50_ values, representing the temperatures required to achieve 90% and 50% methane conversion, respectively. The effects of Mn/Co ratios and gas hourly space velocity (GHSV) on methane oxidation were systematically investigated. As shown in Figure 5a, MnCo_2_O_4.5_ catalysts with varying Mn/Co molar ratios (1:4, 1:5, 1:6, and 1:7) were compared to monometallic Co_3_O_4_ and Mn_3_O_4_. The MnCo_2_O_4.5_-1:5 catalyst exhibited the highest activity, achieving 50% and 90% methane conversion at 326 °C and 395 °C, respectively. In contrast, Co_3_O_4_ and Mn_3_O_4_ required significantly higher temperatures, with T_50_ and T_90_ being 402 °C and 538 °C for Co_3_O_4_ and 467 °C and 581 °C for Mn_3_O_4_, respectively.

Integrated characterization via XPS, BET, O_2_-TPD, and H_2_-TPR revealed that the bifunctional surface nanostructure comprising spherical cores coupled with irregular nanoparticles enhances catalytic activity by exposing additional active sites and improving oxygen mobility. These structural advantages synergistically facilitate methane activation and oxidation kinetics. Figure 5b demonstrates the influence of calcination time on the activity of MnCo_2_O_4.5_-1:5, with the catalyst calcined for 1 h showing superior performance. Stability tests (Figure 5c) confirmed robust durability, retaining 99% of its initial activity after five consecutive cycles.

The impact of GHSV was further assessed (Figure 5d). Increasing GHSV from 24,000 to 64,000 mL·g⁻^1^·h⁻^1^ progressively elevated the T_90_ from 352 °C to 578 °C, indicating a moderate decline in catalytic efficiency under high flow rates. To evaluate practical applicability, the catalyst’s tolerance to water vapor was tested (Figure 5e). Even with 5 vol.% or 10 vol.% H_2_O in the feed gas, the T_90_ remained at 492 °C and 528 °C, respectively, demonstrating exceptional stability with added water. Long-term durability was verified under continuous operation at 400 °C for 20 h (Figure 5f), with negligible activity loss, possessing good thermal stability.

Meanwhile, Figure S3 shows the XRD comparison of MnCo_2_O_4.5_ before and after the reaction. The peak intensity of the material weakens, and the crystallinity decreases after the reaction, which will have an impact on the cyclic performance of the material. MnCo_2_O_4.5_ catalyst exhibits excellent catalytic performance among similar catalysts, even surpassing precious metal-based catalysts (Figure S4), reflecting the advantages of this catalyst in structure and surface composition.

### 3.3. In Situ Raman Measurements

The MnCo_2_O_4.5_ sample was excited with a laser beam (λ = 532 nm). A gas mixture of 1% CH_4_ + 20% O_2_ + N_2_ (with N_2_ serving as the equilibrium shielding gas) was introduced into the Renishaw RM2000 Raman spectrometer, where the mixed gas flow rate was set to 60 mL/min. N_2_ was utilized as an inert gas to flush both the sample and gas path after reaction gas switching. A heating period of 90 min was required for temperature transition between adjacent setpoints. To ensure measurement accuracy, the temperature stabilized for 60 min prior to analysis, followed by Raman characterization conducted for 20 min. During the final 10 min of each temperature stage, the system was heated to the subsequent temperature at a controlled rate of 5 °C/min.

**Figure 6:**
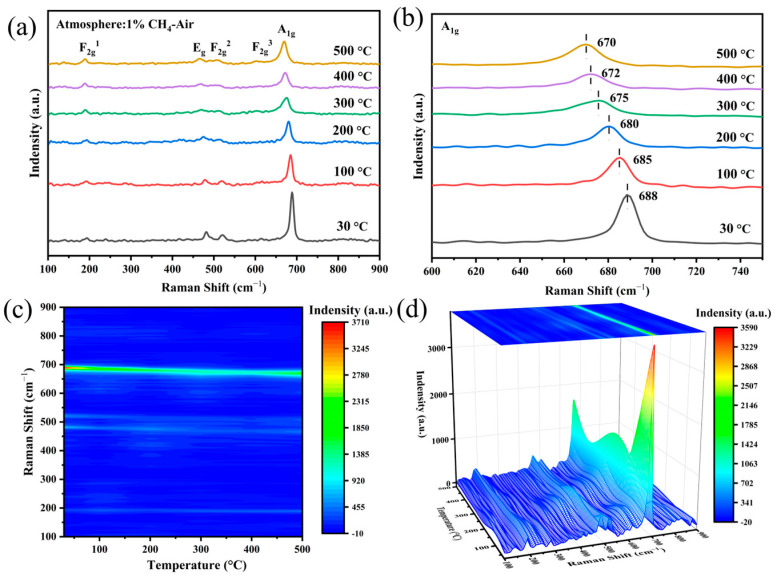
(**a**) In situ Raman spectra of the MnCo_2_O_4.5_ catalyst under a reaction atmosphere of 1% CH_4_ + 20% O_2_ + N_2_ (flow rate: 50 mL·min ^−1^) at varying temperatures. (**b**) Intensity of the A_1g_ peak. (**c**) Contour map corresponding to the in situ Raman spectra. (**d**) Projected 3D mapping surface plot.

In situ Raman experiments were conducted to investigate the evolution of the coordination environment, structural characteristics, and dynamic changes in surface oxygen vacancies during the catalytic reaction process. As shown in Figure 6a, five distinct bands were observed in the range of 100–900 cm^−1^, located at 193, 481, 520, 617, and 688 cm^−1^. Specifically, the band at 193 cm^−1^ corresponds to the F_2g_^1^ Raman-active mode, which is attributed to vibrational modes originating from tetrahedral CoO_4_ units. The peaks at 481 cm^−1^ and 520 cm^−1^ are assigned to the E_g_ and F_2g_^2^ Raman-active modes, respectively, while the weak band at 617 cm^−1^ is associated with the F₂_g_^2^ Raman-active mode. Notably, the intense band at 688 cm^−1^, corresponding to the A_1g_ Raman-active mode, is ascribed to octahedral CoO_6_ units [45,46].

Under 1%CH_4_ + 20%O_2_ + N_2_ atmosphere, a redshift of 18 cm ^−1^ was observed for the A_1g_ mode as the temperature increased from 30 °C to 500 °C (Figure 6b). This redshift is attributed to the optical phonon confinement effect, which induces uncertainty in the phonon wavevector, leading to lattice distortion or residual stress. Consequently, redshift reflects the presence of structural defects. As clearly illustrated in Figure 6c,d, the A_1g_ peak exhibits both redshift and diminished intensity with rising temperature. The negative correlation between A_1g_ peak intensity and temperature suggests the progressive generation of oxygen vacancies on the sample surface. This phenomenon arises because lattice oxygen coordinated with Co^3+^ is released from the crystalline phase to participate in the reaction, while the absence of oxygen replenishment exacerbates the loss of lattice oxygen. The accumulated structural distortion, manifested by the increasing redshift, ultimately destabilizes the octahedral configuration, resulting in a significant reduction in A_1g_ peak intensity. Such structural degradation may further trigger phase transformation under prolonged thermal stress. This behavior underscores the critical role of oxygen vacancy dynamics in modulating the structural stability and catalytic activity of MnCo_2_O_4.5_ under reaction conditions.

## 4. Conclusions

In this work, a series of MnCo_2_O_4.5_ bimetallic catalysts with spherical core-nanoparticle shell structures were designed and synthesized for catalytic methane oxidation. Compared with pure Co_3_O_4_ and Mn_3_O_4_, the MnCo_2_O_4.5_ catalyst exhibited superior activity under lean methane conditions, achieving 90% methane conversion (T_90_) at 395 °C, outperforming most noble metal-based catalysts. The enhanced performance is attributed to the synergistic effects of bimetallic composition and unique hierarchical architecture. The spherical core-irregular surface morphology significantly increases specific surface area, exposing abundant active sites and facilitating rapid oxygen migration. The Mn-Co synergy optimizes electronic structure, promoting redox cycles (Co^3+^/Co^2+^ and Mn^4+^/Mn^3+^) and enhancing lattice oxygen mobility. This dual optimization of geometric and electronic properties possesses efficient methane activation and achieves high catalytic performance. The work provides a novel strategy for designing non-precious metal catalysts through morphology-engineered bimetallic systems, demonstrating great potential for cost-effective methane emission control technologies. The proposed catalyst demonstrates the promising potential for scalable industrial production, attributed to its straightforward experimental methodology. Specifically, carbon spheres can be mass-produced using large-capacity reactors, followed by the adsorption process in a constant-temperature adsorption chamber. Subsequently, the final catalyst products are efficiently manufactured through calcination in tunnel furnaces, enabling high-throughput industrial-scale production.

## Figures and Tables

**Table 1:** XPS results of different catalysts.

Sample	Co 2p			Mn 2p			O 1s		
	Co^3+^	Co^2+^	Co^3+^/Co^2+^	Mn^3+^	Mn^4+^	Mn^3+^/Mn^4+^	O_ads_	O_latt_	O_ads_/O_latt_
MnCo_2_O_4.5_	54.74	45.26	1.21	85.53	14.47	5.91	37.81	62.19	0.61
Co_3_O_4_	29.21	70.79	0.41	-	-	-	45.04	54.96	0.82
Mn_3_O_4_	-	-	-	62.39	37.61	1.66	27.79	72.21	0.38

## Data Availability

Data is contained within the article.

## Supplementary Materials

The following are available online at https://www.mdpi.com/article/10.3390/nano15070524/s1, Text S1: Preparation of Co_3_O_4_ catalyst and Preparation of Mn_3_O_4_ catalyst; Figure S1: XRD of samples with different calcination times with calcination temperature at 500 ^o^C; Figure S2: SEM images of the different samples; Figure S3: XRD of samples before and after testing; Figure S4: CH_4_ oxidation activity for different catalysts; Table S1: BET results of different catalysts.
