# Supplementary material for: Rich Oxygen Vacancies in Bimetallic MnCo2O4.5 Spheres for Enhancing Lean Methane Catalytic Oxidation
Source: Nanomaterials (Basel). 2025 Mar 31;15(7):524. doi: 10.3390/nano15070524 (PMC11990540; doi:10.3390/nano15070524)
Supplement: Supplementary file 1 [file nanomaterials-15-00524-s001.zip › nanomaterials-3542570-supplementary.pdf]

Supplementary Information

Rich Oxygen Vacancies in Bimetallic $MnCo_2O_{4.5}$ Spheres for Enhancing Lean Methane Catalytic Oxidation

Ke Yang[a], Chenqi Li[a], Qinghan Zhu[a], Haiwang Wang[a]*, Jian Qi[b,c]*

[a] Key Laboratory of Dielectric and Electrolyte Functional Material Hebei Province, Northeastern University at Qinhuangdao, Qinhuangdao, 066004, China

[b] State Key Laboratory of Biochemical Engineering, Institute of Process Engineering, Chinese Academy of Sciences, Beijing, 100190, China

[c] School of Chemical Engineering, University of Chinese Academy of Sciences, Beijing, 100049, China

[c] Corresponding authors.

E-mail: whwdbdx@126.com (H. Wang), jqi@ipe.ac.cn (J. Qi)

Text S1

Preparation of Co_3O_4 catalyst

Synthesis of Co_3O_4 catalyst: A mixture of 0.60 g sucrose carbon spheres,3.49 g cobalt nitrate, and 3 g urea were dissolved in a solution of 15 mL water and 45 mL ethanol, then ultrasonicated for 15 minutes. The suspension was heated in a Teflon-lined autoclave at 80 °C for 6 h, followed by washing with deionized water and ethanol, and drying overnight at 80 °C. The dried precursor was then calcined in air at 500 °C for 1 h with a heating rate of 1 °C/min.

Preparation of Mn_3O_4 catalyst

Synthesis of Mn_3O_4 catalyst: A mixture of 0.60 g sucrose carbon spheres, 2.94 g manganese acetate and 3 g urea were dissolved in a solution of 15 mL water and 45 mL ethanol, then ultrasonicated for 15 minutes. The suspension was heated in a Teflon-lined autoclave at 80 °C for 6 h, followed by washing with deionized water and ethanol, and drying overnight at 80 °C. The dried precursor was then calcined in air at 500 °C for 1 h with a heating rate of 1 °C/min.

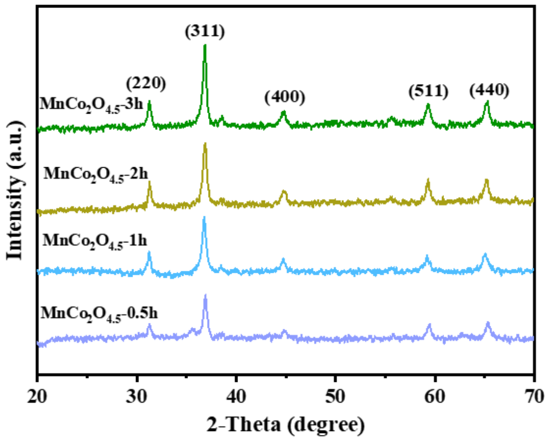

Figure S1. XRD of samples with different calcination times with calcination temperature at 500 °C

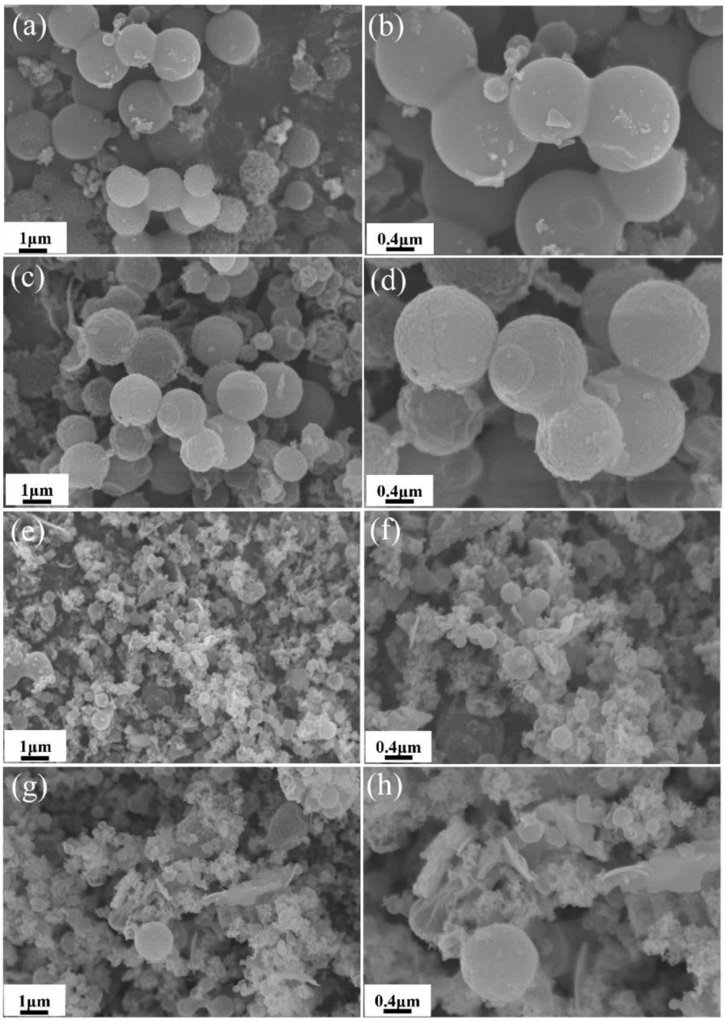

Figure S2. SEM images of the different samples
(a-b) $MnCo_2O_{4.5}$-0.5h; (c-d) $MnCo_2O_{4.5}$-1h; (e-f) $MnCo_2O_{4.5}$-2h; (g-h) $MnCo_2O_{4.5}$-3h

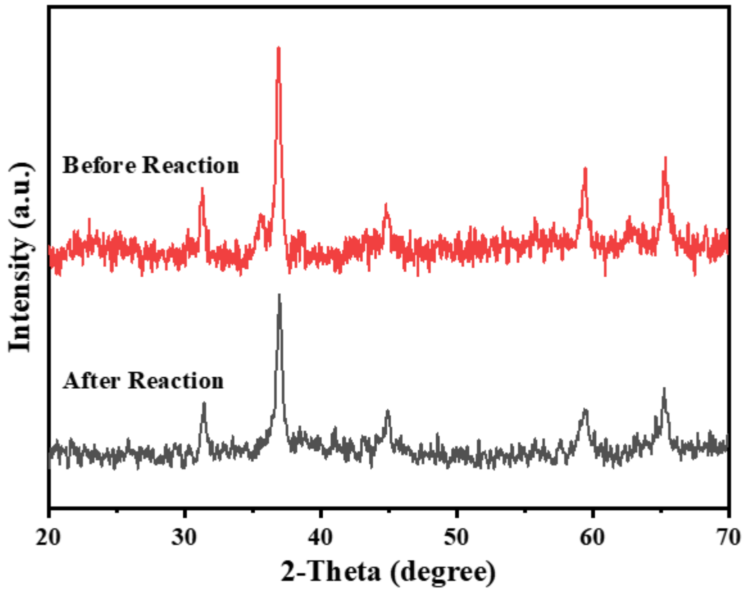

Figure S3. XRD of samples before and after testing

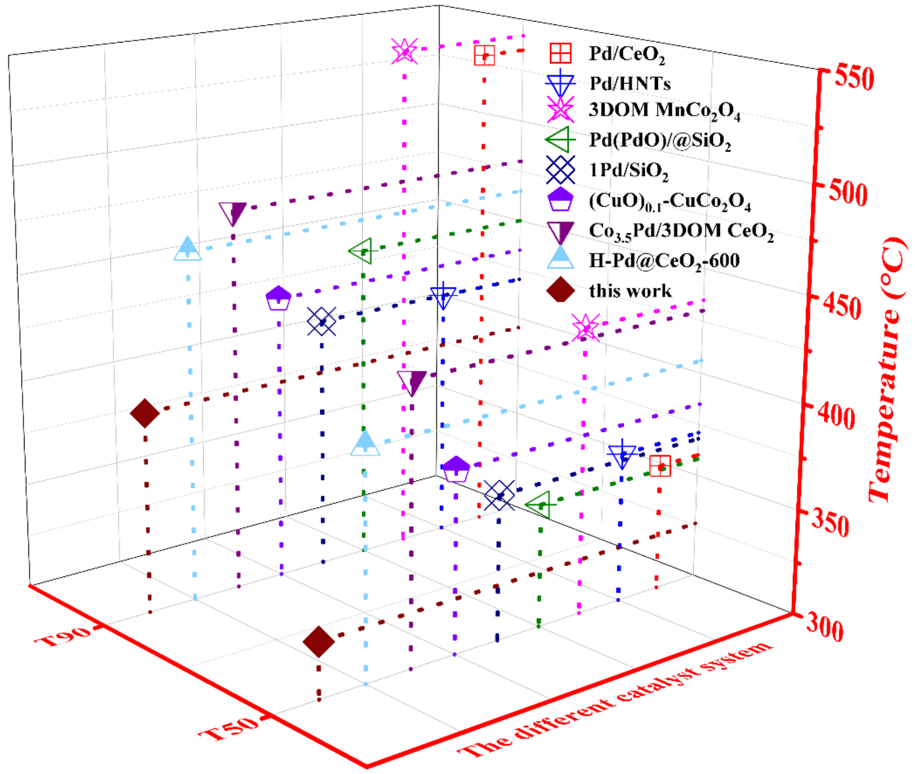

Figure S4. CH_4 oxidation activity for different catalysts [S1-S8].

Table S1. BET results of different catalysts

Sample	specific surface area (m^2/g)	pore volume (cm^3/g)	Average pore size (nm)
$MnCo_2O_{4.5}$	56.58	0.519	11.47
Co_3O_4	41.14	0.610	2.97
Mn_3O_4	13.09	0.052	20.04

References

S1. Ahmad, Y.H.; Mohamed, A.T.; Al-Qaradawi, S.Y. Exploring halloysite nanotubes as catalyst support for methane combustion: Influence of support pretreatment. *Applied Clay Science* **2021**, *201*, 105956, doi: 10.1016/j.clay.2020.105956.

S2. Han, Z.; Dai, L.; Liu, Y.; Deng, J.; Jing, L.; Zhang, Y.; Zhang, K.; Zhang, X.; Hou, Z.; Pei, W. AuPd/Co_3O_4/3DOM $MnCo_2O_4$: Highly active catalysts for methane combustion. *Catalysis Today* **2021**, *376*, 134-143, doi: 10.1016/j.cattod.2020.06.068.

S3. Ma, Y.; Li, S.; Zhang, T.; Zhang, Y.; Wang, X.; Xiao, Y.; Zhan, Y.; Jiang, L. Construction of a Pd (PdO)/Co_3O_4@SiO_2 core-shell structure for efficient low-temperature methane combustion. *Nanoscale* **2021**, *13*, 5026-5032, doi: 10.1039/d0nr08723h.

S4. Chen, S.; Chen, R.; Su, Z.; Wang, H.; Zhang, X.; Chen, T. Constructing of Mn^{4+}-O-

PdO_x sites in $SmMn_2O_5$ Mullite: Novel and Highly-Active reactive centers for Low-Temperature methane combustion. *Journal of Catalysis* **2023**, *425*, 8-19, doi: 10.1016/jjcat.2023.06.005.

S5. Shao, X.; He, J.; Su, Q.; Zhao, D.; Feng, S. Synergy effect of CuO on $CuCo_2O_4$ for methane catalytic combustion. *RSC Advances* **2022**, *12*, 17490-17497, doi: 10.1039/D2RA02237K.

S6. Xie, S.; Liu, Y.; Deng, J.; Zhao, X.; Yang, J.; Zhang, K.; Han, Z.; Dai, H. Three-dimensionally ordered macroporous CeO_2-supported Pd@Co nanoparticles: Highly active catalysts for methane oxidation. *Journal of Catalysis* **2016**, *342*, 17-26, doi: 10.1016/j.jcat.2016.07.003.

S7. Cai, G.; Luo, W.; Xiao, Y.; Zheng, Y.; Zhong, F.; Zhan, Y.; Jiang, L. Synthesis of a highly stable Pd@CeO_2 catalyst for methane combustion with the synergistic effect of urea and citric acid. *ACS Omega* **2018**, *3*, 16769-16776, doi: 10.1021/acsomega.8b02556.s001.

S8. Zheng, Y.; Yu, Y.; Zhou, H.; Huang, W.; Pu, Z. Combustion of lean methane over Co_3O_4 catalysts prepared with different cobalt precursors. *RSC Advances* **2020**, *10*, 4490-4498, doi: 10.1039/c9ra09544f.
